# Cytotoxic Effects and Micronuclei Frequency as a Biomarker of Genotoxicity in Farmers from the Municipality of Tehuacán, Puebla, Mexico

**DOI:** 10.3390/toxics13090735

**Published:** 2025-08-30

**Authors:** Amparo Mauricio-Gutiérrez, Didier D. Ramírez-Gutiérrez, Omar Romero-Arenas, Carlos A. Contreras-Paredes, Sandra Mora-Ravelo, Lilia Cedillo-Ramírez, José A. Yáñez-Santos, María A. Valencia de Ita

**Affiliations:** 1 Secretaría de Ciencia, Humanidades, Tecnología e Innovación-Centro de Agroecología, Instituto de Ciencias, Benemérita Universidad Autónoma de Puebla, Edificio VAL 1, Km 1.7 Carretera a San Baltazar Tetela, San Pedro Zacachimalpa 72960, Puebla, Mexico; amg2510@hotmail.com; 2 Facultad de Ciencias Biológicas, Benemérita Universidad Autónoma de Puebla, Puebla 72570, Puebla, Mexico; dayron.ramirez@alumno.buap.mx; 3 Centro de Agroecología, Instituto de Ciencias, Benemérita Universidad Autónoma de Puebla, Edificio VAL 1, Km 1.7 Carretera a San Baltazar Tetela, San Pedro Zacachimalpa 72960, Puebla, Mexico; mavi1179@outlook.es; 4 Jardín Botánico Universitario, Benemérita Universidad Autónoma de Puebla, Prolongación de la 24 Sur y Av. San Claudio, Ciudad Universitaria. Col. San Manuel, Puebla 72570, Puebla, Mexico; carlos.contreraspar@correo.buap.mx; 5 Facultad de Ingeniería y Ciencias, Universidad Autónoma de Tamaulipas, Campus Tamaulipas, Centro Universitario, Ciudad Victoria 87149, Tamaulipas, Mexico; sgmora@docentes.uat.edu.mx; 6 Centro de Investigación en Ciencias Microbiológicas, Instituto de Ciencias, Benemérita Universidad Autónoma de Puebla, Puebla 72570, Puebla, Mexico; lilia.cedillo@correo.buap.mx; 7 Posgrado en Ciencias Ambientales, Instituto de Ciencias, Benemérita Universidad Autónoma de Puebla, Puebla 72570, Puebla, Mexico; 8 Centro de Detección Biomolecular, Vicerrectoría de Investigación y Estudios de Posgrado, Benemérita Universidad Autónoma de Puebla, Puebla 72570, Puebla, Mexico

**Keywords:** micronuclei cells, pesticides, rural population

## Abstract

In Tehuacán, Puebla, Mexico, the agricultural sector is primarily dedicated to corn cultivation, which is reflected in the region’s economy, culture, and diet. This sector follows an agro-industrial production model dependent on pesticides and chemical fertilizers, which impacts both soil health and the population’s well-being. The objective of this study was to assess cytotoxic damage using the Buccal Micronucleus Cytome Assay (BMCA) in a population engaged in agricultural activities in San Diego Chalma, Tehuacán, Puebla, Mexico. Sociodemographic parameters were analyzed, along with the buccal micronucleus cytome assay, in a sample of 35 individuals composed of an agricultural group (18) and a control group (17). The agricultural group showed a significantly higher number of total micronucleated cells (Median = 714), which was 19.8 times greater than the non-agricultural group. Age, sex, basic education level, time of residence, and involvement in agricultural activities were key factors contributing to the development of buccal cell micronuclei, in addition to the use of pesticides as lambda-cyhalothrin, spinetoram, ethoprophos, carbofuran, methomyl, and chlorpyrifos ethyl without safety measures. There was an increased risk of developing micronucleated cells in males from the control group (OR = 2.386, 95% CI = 2.123–2.681) and in individuals aged 30–59 years (OR = 16.464, 95% CI = 14.315–18.935). The agricultural population for the 0–29 years presented a risk probability developing micronucleated cells of 99.8% in men and 99.9% in women, with a higher risk observed in women and in individuals who had lived their entire lives in San Diego Chalma, where they are continuously exposed to pesticides. Therefore, it is crucial to provide guidance, training, and improved public policies in the region of Puebla, Mexico.

## 1. Introduction

In Mexico, 7.76% of the population is engaged in agricultural activities [1]. Corn is one of the predominant crops, accounting for 34.66% of the country’s total cultivated area, with 6.9 million hectares sown; 7.40% of that total is contributed by the state of Puebla, Mexico, where corn is one of the most widely grown grasses, representing 55.96% of the cultivated area [2]. In addition, corn production in the municipality of Tehuacán, Puebla contributed 48.28% of the state’s output, highlighting its economic, nutritional, and cultural importance in the region [3].

The primary sector’s participation in corn cultivation in Tehuacán, Puebla, Mexico follows an agro-industrial production model that relies heavily on external inputs such as pesticides and chemical fertilizers, which has significant implications for the sustainability of agroecosystems [4].

In Mexico, over the last two decades, total pesticide consumption has increased by 57–65% [5], along with fertilizer use (approx. 2,200,000 tons), both of which are common in conventional agricultural systems [6]. The intrinsic toxicity of pesticides poses a risk to farmers, rural populations, and organisms within agroecosystems.

The handling and application of pesticides pose a risk to humans, whether as users or consumers of treated vegetables, fruits, and other products [7]. In Mexico, there is a list of prohibited and restricted pesticides due to their high risk to human health and their high persistence and bioaccumulation properties, which continue to be used, such as: lambda-cyhalothrin, spinetoram, ethoprophos, carbofuran, methomyl, and chlorpyrifos ethyl [8].

Some pesticides have been shown to have cytotoxic effects in vivo in animals, i.e., category 2 and 3 mutagens, for example, benomyl, carbendazim, DNOC (4,6-dinitro-o-cresol), methyl bromide, monocrotophos, phosphamidon) and others considered devoid of cytotoxic activity [7]. However, subsequent studies found different concentrations of pesticides in human adipose tissue such as p,p’-DDE (~1.65 mg/kg); p,p’-DDT (0.23 mg/kg) and o,p’DDT (0.022 mg/kg), with higher concentrations being detected in the elderly population [9].

Monitoring the different pesticides mentioned by farmers will allow for the creation of human health risk models. This will allow for the implementation of better management practices and safety policies for the most used pesticides, and for the implementation of good agricultural practices in their use, handling, and containment. Exposure to pesticides in agricultural practices represents a significant public health problem, coupled with a lack of knowledge and adequate training on the safe use of pesticides. Therefore, it is crucial to develop and use accurate indicators to assess these toxicological risks and reduce vulnerability to cytotoxic damage.

Moreover, their cytotoxic potential is a primary risk factor and has been evaluated using the Buccal Micronucleus Cytome Assay (BMCA), a method that utilizes peripheral lymphocytes or exfoliated buccal mucosa cells to assess long-term toxicological effects related to carcinogenesis, reproductive health, and degenerative conditions [10].

One of the biological indicators of this assay is the presence of micronuclei, which are chromosomal fragments or whole chromosomes formed during anaphase. These fragments fail to integrate into the nuclei of daughter cells and instead form one or more micronuclei in the cytoplasm [11]. The BMCA of buccal cells is a biomarker of genetic damage caused by lifestyle habits such as tobacco and alcohol consumption, micronutrient deficiencies, pesticide exposure, and hereditary genetic defects [12]. It is a non-invasive technique, making it an attractive option for biomonitoring human populations or individuals [13]. Therefore, the aim of this study was to assess cytotoxic damage through BMCA monitoring in a population engaged in agricultural activities in San Diego Chalma, Tehuacán, Puebla, Mexico.

## 2. Materials and Methods

### 2.1. Study Area

**Figure 1:**
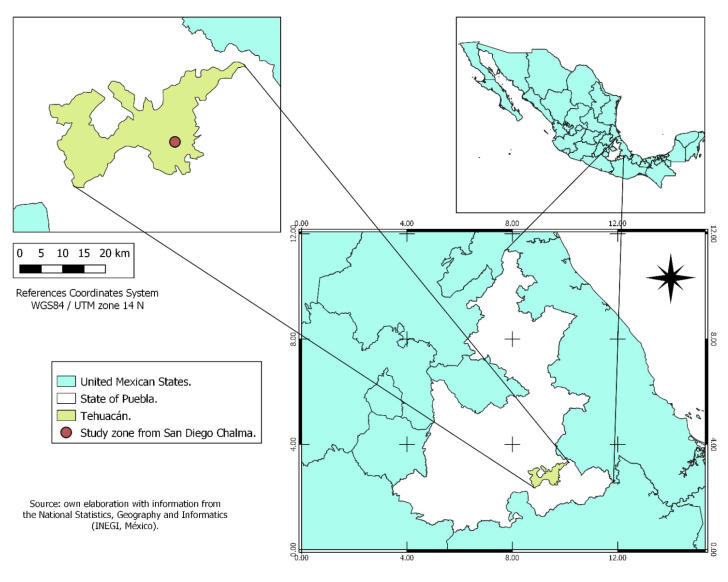
Location of study zone in the municipality from San Diego Chalma, Tehuacán, Puebla, Mexico.

The study site is in San Diego Chalma, Tehuacán, Puebla, Mexico, at geographic coordinates 18°26′13.9992″ N and 97°21′41.0004″ W (Figure 1). The average annual temperature ranges between 18 °C and 22 °C, with annual precipitation between 200 and 1800 mm [14].

### 2.2. Sample Size Selection

The study population consisted of residents of San Diego Chalma and the city of Tehuacán, Puebla, Mexico. The sample included a group of 18 farmers and a control group of 17 individuals with no connection to agricultural activities. The total sample size was 35 participants, which exceeded the minimum sample size calculated using Santoyo’s formula [15]. A simple random sampling design was used, with a two-tailed alpha (d) of 0.05, a normal distribution (z) value of 1.96, and a coefficient of variation (CV) of 0.8, resulting in a required size (n) of 28. The formula used to calculate the sample size considering two curricula or treatments (local farmers and control group) was Equation (1):

*n* = (N × Z^2^ × CV^2^)/[((N − 1) × *d*^2^) + (Z^2^ × CV^2^)] (1)

where

N = Represents the total number of producers;

Z = Normal distribution;

CV = Coefficient of variability;

*d* = Bilateral alpha;

*n* = Sample size.

### 2.3. Buccal Micronucleus Cytome Assay (BMCA)

The Buccal Micronucleus Cytome Assay was used as a biomarker of cytotoxicity, including the assessment of nuclear anomalies in randomly selected farmers from the region. A gentle buccal smear of the oral mucosa on both cheeks was collected using sterile swabs, in accordance with the Declaration of Helsinki [12]. One sample per person was collected and stored in 1 mL of saline solution (0.9% NaCl) at 4 °C until further analysis.

Each sample was centrifuged at 1500 rpm for 10 min, and the cellular sediment was spread across 10 microscope slides. The slides were air-dried at room temperature for 10 min and stained using the Feulgen method. This involved fixing the cells with Farmer’s fixative solution (75% absolute ethanol and 25% glacial acetic acid) for 15 min, followed by washes with 70% ethanol and distilled water. After drying for 5 min, 1N HCl was applied for 45 min at room temperature for hydrolysis. The slides were rinsed with distilled water and stained using a carmine-based dye (carmine-propionic or acetocarmine solution: 1 g carmine in 100 mL of 45% glacial acetic acid) for 30 min or until the tissue appeared purple. After another rinse with distilled water and drying, the samples were examined under a compound microscope (Carl Zeiss, Jena, Germany) at 10× and 40× magnification. The frequency of micronucleated cells (MN), nuclear buds (BUD), and binucleated cells (BN) was determined as indicators of potential aneuploidy and genomic instability. Additional indicators such as condensed chromatin cells (CC), karyorrhectic cells (KR), karyolytic cells (KL), pyknosis (PY), and basal cells (BC) were analyzed to assess epithelial proliferation activity in the buccal mucosa [16].

### 2.4. Statistical Analysis

Sociodemographic variables including age, sex, place of birth, duration of residence, education level, occupation, alcohol and tobacco use, and infectious diseases were analyzed. Pearson’s Chi-square (χ^2^) test was performed with a significance level of 0.05 to compare the farmer and non-farmer groups. Additionally, the use of pesticides and the implementation of safety measures during preparation, application, and waste storage were investigated.

Associations between sociodemographic factors and the presence of micronucleated cells (MN) in the study population were also examined using Pearson’s Chi-square test (*p* ≤ 0.05). The Shapiro–Wilk test was applied to verify the normality of the variables, followed by the Mann–Whitney U test (*p* ≤ 0.05) for independent samples to determine statistically significant differences in the total number of micronuclei (MNi), condensed chromatin cells (CC), karyorrhectic cells (KR), binucleated cells (BN), karyolytic cells (KL), pyknosis (PY), micronucleated cells (MN), and basal cells (BC) between the farmer group (F) and the non-farmer group (NF).

Finally, a multinomial analysis was conducted using a generalized linear model to predict the development of micronucleated cells (MN) with predictive variables including age, place of birth, and sex of the non-agricultural population. Odds ratios (Exp B) were calculated to express the association between the outcome and the explanatory variables as multivariate risk coefficients for the formation of micronucleated cells. Statistical significance was determined at a 95% confidence interval (CI) and *p*-values ≤ 0.05. All calculations were performed using SPSS Statistics version 17 for Windows.

## 3. Results

### 3.1. Sociodemographic Parameters

The study area included a group of farmers (18 individuals) engaged in agricultural activities (100%) and a group of non-farmers (17 individuals), mainly involved in commerce (58%; *p* < 0.001). The population studied (Table 1) was within the 30–59 age range (*p* = 0.632), and 70% were men (*p* = 0.621). Among the farmers, 61% were born in San Diego Chalma, while 70% of the non-agricultural population were born in the city of Tehuacán, Puebla, Mexico (*p* = 0.06). Additionally, 55% of farmers had lived in San Diego Chalma for over 30 years, compared to 30% of the control group, with no significant differences (*p* = 0.092). Both groups had a basic education level, with a higher percentage among farmers (72%) than in the control group (35%).

However, 39% of farmers reported tobacco use (1–5 cigarettes per week), and 61% consumed alcohol once a week. In contrast, only 12% and 29% of the control group reported tobacco and alcohol use, respectively. No significant differences were found between the two groups (*p* = 0.774) regarding infectious disease occurrence.

Six insecticide-type pesticides were identified: lambda-cyhalothrin, spinetoram, ethoprophos, carbofuran, methomyl, and chlorpyrifos ethyl [7,8]. These active ingredients belong to various toxicological categories (Ib, II, III, IV), with lambda-cyhalothrin, methomyl, and chlorpyrifos ethyl applied at a frequency of over 20% by regional farmers, typically without using protective equipment during preparation or application, especially in corn crops. Moreover, irrigation with wastewater from the textile industry further endangers their health (Table 2).

### 3.2. Chromosomal Aberration Counts

Descriptive analysis of the cellular aberration counts revealed that micronucleated cells (MN) were the most frequent in both groups, with a significantly higher frequency (34,545) in the farmer group compared to the control group (4189) (*p* < 0.001). Age influenced the increase in micronuclei in both groups (*p* < 0.001), with farmers over 30 years old showing more micronucleated cells (24,827), while the reference group had 3966 MNs. Male farmers had more micronucleated cells (27,450) than females (7095), both significantly higher than the control group (*p* < 0.001). This activity compromises producers’ health by increasing the number of MNs (34,545) (Table 3).

**Figure 2:**
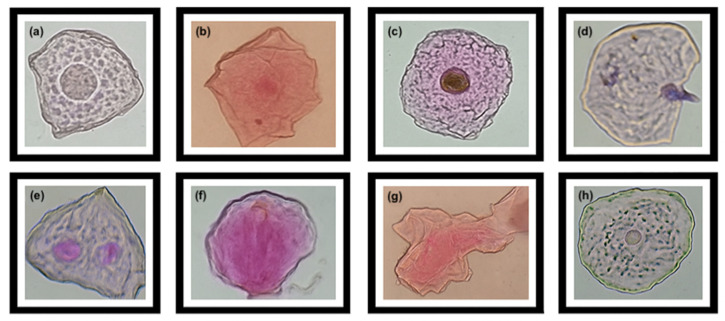
Cells with nuclear aberrations due to cytotoxic damage in the farming group. (**a**,**b**): micronuclei (MN); (**c**): condensed chromatin (CC); (**d**): karyorrhexis (KR); (**e**): binucleate cells (BN); (**f**): karyolysis (KL); (**g**): pyknosis (PY); (**h**): basal cells (BC).

**Figure 3:**
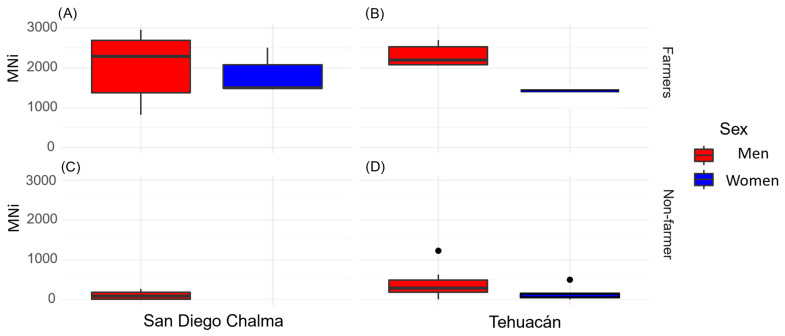
Total number of micronuclei cells (MNi) in the farming group ((**A**): birthplace in San Diego Chalma; (**B**): birthplace in Tehuacán)) and non-farmer (**C**): birthplace in San Diego Chalma; (**D**): birthplace in Tehuacán)).

Residents living more than 30 years in San Diego Chalma had more micronuclei aberrations, indicating that prolonged exposure to contaminants such as pesticides caused adverse effects (Figure 2 and Figure 3). Additionally, education level influenced the number of MNs, with those having only primary education showing the highest counts: 25,762 in the farmer group versus 1403 in the control group.

Tobacco and alcohol use and infectious diseases were not associated with MN presence according to the chi-square test conducted on the studied population (Table 3).

The Buccal Micronucleus Cytome Assay revealed a significantly different (*p* ≤ 0.05) presence of epithelial cells with abnormalities between the two groups (Table 4; Figure 2 and Figure 3). The farmer group (F) showed a significantly higher total number of micronuclei (MNi) (Median = 714; U = 2,600,576; *d* = 6.17) than the non-agricultural population (Median = 36; *p* < 0.001). Highlighting the male sex who was born in San Diego Chalma dedicated to agricultural activities with a greater number of micronuclei (Figure 3). The most notable abnormalities included karyolytic cells (KL) (Median = 2096; U = 0; *d* = 2.88), pyknosis (PY) (Median = 1129; U = 1,834,096; *d* = 2.65), and micronucleated cells (MN) (Median = 2581; U = 0; *d* = 3.39). Additionally, the farmer group (F) had significantly fewer basal cells (Median = 2792; U = 757,872; *d* = 13.86) than the control group (Median = 9501, *p* < 0.001).

A multinomial analysis of sociodemographic factors such as age, place of birth, and sex with MNs in both groups (Table 5) showed that the generalized linear model was statistically significant (*p* < 0.001; Cox and Snell R^2^ = 16.8%, Nagelkerke R^2^ = 33.9%), indicating a strong explanatory power of risk-related conditions for the development of micronucleated cells.

In the control group (non-agricultural), the development of abnormal epithelial cells was significantly related to age (*p* < 0.001), birthplace (*p* < 0.001), and sex (*p* < 0.001). Individuals aged 30–59 had 16 times the likelihood of developing MNs (OR = 16.464; 95% CI = 14.315–18.935; *p* < 0.001) compared to younger populations. While birthplace was significantly associated with MN development (*p* < 0.001), it did not increase the probability of developing MNs (OR = 0.229; 95% CI = 0.217–0.240; *p* < 0.001), regardless of whether the individual was born in Tehuacán or San Diego Chalma. However, sex was significantly related to MNs (*p* < 0.001), with males having twice the likelihood of cytotoxic damage (OR = 2.386; 95% CI = 2.123–2.681; *p* < 0.001) (Table 5).

According to the model generated in Table 5, the results obtained showed that exposure to pollutants (pesticides) in the agricultural population who had lived their entire lives in San Diego Chalma caused changes in cytokinesis and led to a higher number of micronucleated cells for the 0–29 age range, with a probability of 99.8% in men and 99.9% in women; as well as for the 30–59 age group, with a probability of 97.1% and 98.8% in men and women, respectively, related to cytotoxic damage (Table 6). On the other hand, the agricultural population born in Tehuacán had a risk of developing micronucleated cells of 96.7% in men and 98.6% in women in the 0–29 age range; The population aged 30–59 years showed a risk probability of developing micronucleated cells of 64.0% (men) and 80.9% (women). It is noteworthy that women engaged in agricultural activities were at higher risk of developing micronucleated cells. It is worth mentioning that in Mexico, the male population is primarily dedicated to field work, although women are less likely to participate, and they are more likely to develop cellular abnormalities (MN).

On the other hand, in the reference group of both men and women from San Diego Chalma, the risk probability of developing micronucleated cells was very low (0.2–0.1%) in the 0–29 age range. Cytotoxic damage is age-related, as the number of MN tends to increase for people aged 30–59 years, mainly in men (2.9% probability) compared to women (1.2% probability). Furthermore, the population born in Tehuacán and not engaged in agricultural activities showed the same cytotoxic damage behavior positively correlated with age (30–59 years) with a risk probability of 36.0% in men and 19.1% in women.

Agricultural activities in San Diego Chalma expose the population to pesticides due to lack of protective equipment—mostly due to discomfort and lack of awareness about health hazards during preparation, application, and storage—which poses a significant risk for developing micronucleated cells.

## 4. Discussion

Corn is susceptible to phytopathological problems caused mainly by diseases from the Fusarium and Aspergillus genera, which lead to root, stalk, and ear rot. These diseases result in economic losses due to low yields, compromising food security and affecting the health of both producers and consumers in the region due to the heavy reliance on pesticide use [17].

According to the present study, the pesticides Lambda-Cyhalothrin, Methomyl, and Chlorpyrifos ethyl were the most frequently used by agricultural producers in San Diego Chalma, Tehuacán, Puebla, primarily for corn cultivation (Table 2). These pesticides fall under toxicological categories II and Ib [7]. Therefore, it is crucial to generate data on the cytotoxic risk posed by local agricultural practices in San Diego Chalma to raise awareness and help prevent public health implications due to the improper use of pesticides. Farmers in the region do not use any protective equipment during pesticide preparation, application, or waste management—this practice is common throughout Mexico and is compounded by low to medium levels of education [10].

Pesticide exposure represents a major public health issue [18] that has persisted since the Green Revolution [19]. The lack of knowledge and proper training on the safe use of pesticides has led to intensive agriculture practices that negatively impact both soil health and human health [20,21].

Cytotoxicity testing has been widely reported as a means of assessing DNA damage, including single- and double-strand breaks, point mutations, chromosomal aberrations, and micronucleus formation, among others [22]. In this study, Lambda-Cyhalothrin has been shown to cause genomic instability and cytotoxic effects—such as a significant increase in micronucleus frequency and apoptosis—in Chinese hamster ovary (CHO-K1) cells at doses ranging from 10 to 100 µg/mL [23]. Similarly, Methomyl and Chlorpyrifos ethyl have been associated with increased cytotoxic effects in human testicular and liver cells [24,25].

For this reason, the Buccal Micronucleus Cytome Assay was crucial in assessing risk in the exposed population at the study site, enabling the implementation of better pesticide handling practices and safety policies in Mexico. The agricultural population of San Diego Chalma showed evidence of cytotoxic damage, primarily reflected in the number of micronucleated cells (Median = 2581), as well as other types of cellular anomalies such as karyolytic (KL) cells (Median = 2096) and pyknosis (PY) (Median = 1129) (Table 4). Age, sex, education level, duration of residence, and involvement in agricultural activities were significant factors for the development of micronuclei in buccal cells, compromising the health of local farmers. However, habits such as tobacco or alcohol use and infectious diseases were not associated (Table 3).

These findings are consistent with Rivera et al. [10], who also found no association between micronucleated cells and tobacco or alcohol use. However, their study did find differences in total micronucleated cells between conventional (408) and agroecological (253) farming practices, with an emphasis on CC (karyorrhexis) and KL (karyolytic) cells. In contrast, our study found the total proportion of micronucleated cells was 1.9 times higher than that observed in conventional farmers [10], and 19.8 times higher compared to individuals not engaged in farming activities.

The risk level for developing micronucleated cells in the reference group (non-farmers) was associated with the 30–59 age range (OR = 16.464, 95% CI = 14.315–18.935) and male sex (OR = 2.386, 95% CI = 2.123–2.681) (Table 5). However, the farming population exhibited the highest number of micronucleated cells, with a risk probability ranging from 64% to 99.9%, especially among women and individuals born and raised in San Diego Chalma (Table 6).

Micronuclei are not only indicators of chromosomal damage, but can also significantly affect cell functionality, cause genomic instability and chromosomal rearrangements (e.g., aneuploidy, reintegration of damaged DNA fragments, chromothripsis), and activate DNA damage response mechanisms (ATM/ATR: Ataxia Telangiectasia Mutated and Rad3-Related Kinases), senescence, apoptosis, and immune and inflammatory responses via the cGAS-STING pathway [26].

Therefore, the agricultural population of San Diego Chalma, Tehuacán, Puebla, experiences greater cytotoxic damage than the population studied in Españita, Tlaxcala, Mexico [10]. In both studies, farmers did not use safety or protective equipment during irrigation, preparation, or pesticide application—identified as a risk factor by Ferré et al. [27], who reported that 65% of farmers use only one to three personal protective elements. In contrast, our study found that no more than three items of protective gear were used (boots, gloves, and mask), underscoring the need for training and guidance on pesticide use to reduce their vulnerability to cytotoxic harm.

The issue of pesticides presents a multifaceted challenge, with intertwined environmental and health impacts that demand urgent attention and innovative solutions. The findings highlight the urgent need for a transition toward green agriculture approaches that reduce dependence on chemical pesticides and promote safer, more sustainable practices. Green agriculture integrates agroecological strategies such as crop rotation, biological control, biofertilizers, and organic amendments, which minimize environmental and health risks for producers while maintaining agroecosystem productivity. Recent studies emphasize that adopting integrated pest management (IPM) and agroecological practices can significantly mitigate cytotoxic risks and improve soil–plant–human health interactions. Public awareness and education are essential components for fostering collective commitment to green agriculture. Governments, researchers, and stakeholders must collaborate to enact and enforce strict regulations that prioritize the development and adoption of ecological solutions for food production [28].

In this context, it is necessary to implement community-level interventions to prevent pesticide poisoning and cellular damage among agricultural producers. Therefore, the following measures are proposed:

1. Training programs for farmers on the safe handling, preparation, and application of pesticides, with emphasis on the risks of chronic exposure and DNA damage.
2. Mandatory use of personal protective equipment (PPE), including gloves, boots, protective masks, coveralls, and safety goggles, to reduce dermal and inhalation exposure.
3. Promotion of integrated pest management (IPM) practices, replacing highly hazardous pesticides with biological control agents, resistant crop varieties, and essential oils with fungicidal and insecticidal properties.
4. Establishment of safe storage and disposal systems for pesticides and their residues to prevent contamination of soil, water, and household environments.
5. Community monitoring and biomarker surveillance programs, including periodic cytotoxicity testing (e.g., BMCA), to detect early genetic damage and support evidence-based health policies.

By incorporating these strategies within a green agriculture framework, it is possible to reduce cytotoxic risk, improve public health outcomes, and promote the long-term sustainability of rural communities in San Diego Chalma and similar agricultural regions [10].

## 5. Conclusions

Oral cytome samples collected in the community of San Diego Chalma, Tehuacán, Puebla, showed chromosomal aberrations (micronucleated cells, condensed chromatin, karyorrhexis, binucleated cells, karyolysis, and pyknosis) with increased cytotoxic risk in the agricultural population due to exposure to pesticides without adequate protection and safety measures. Therefore, it is necessary to provide advice and training and improve public policies on the use of pesticides in agricultural communities in the Puebla region, Mexico.

## Figures and Tables

**Table 1:** Social and health indicators in study zone.

Variables	Category	Farmers Group		Non-Farmer Group		*p* Value
		F = 18		NF = 17		
		Frequency	%	Frequency	%	
Age	0–29 years	5	27.8	6	35.3	0.632
	30–59 years	13	72.2	11	64.7	
Sex	Women	4	22.2	5	29.4	0.621
	Men	14	77.8	12	70.6	
Birthplace	Tehuacán	7	38.9	12	70.6	0.060
	San Diego Chalma	11	61.1	5	29.4	
Time of residence in the town	0–5 years	2	11.1	3	17.6	0.092
	6–10 years	1	5.6	1	6.0	
	10–20 years	4	22.2	3	17.6	
	21–30 years	1	5.6	5	29.4	
	>30 years	10	55.5	5	29.4	
Education level	Elementary education	13	72.2	6	35.3	0.179
	Secondary education	2	11.1	3	17.6	
	Higher Secondary education	2	11.1	3	17.6	
	Technical education	0	0	3	17.6	
	No education	1	5.6	2	11.8	
Occupation	Farmer	18	100	0	0	0.001 **
	Businessman	0	0	10	58.8	
	Housewife	0	0	2	11.8	
	Student	0	0	3	17.6	
	Minors	0	0	2	11.8	
Smoker	1–5 cigarettes/week	7	38.9	2	11.8	0.067
	No smoking	11	61.1	15	88.2	
Alcohol consumer	2 to 3 times a week	1	5.6	1	5.9	0.158
	Once a week	11	61.1	5	29.4	
	No alcohol	6	33.3	11	64.7	
Diseases Infections	Once a year	5	27.8	4	23.5	0.774
	None	13	72.2	13	76.5	

F: People of farmers; NF: People of non-farmers. **: highly significant.

**Table 2:** Pesticides used by producers in San Diego Chalma Tehuacán, Puebla.

Ingredient Active	Chemical Family	Commercial Name	Type ^1^	Mechanism of action	TC ^2^	List HHP ^3^	Pesticide Application
Lambda-Cyhalothrin	Pyrethroids	Lambda Cihalotrina 50 EC, Lambda KOOR 5.6%, LambdaTel 70CE, Lambdazo	I	Affects sodium channels in nerve cells	III	1, 3	33.33% (6/18)
Spinetoram	Spinosines	Palgus, Exalt 60SC, Spirotrid	I	Activates nicotinic acetylcholine receptors causing paralysis	IV	1, 2, 3	16.66% (3/18)
Ethoprophos	Organophosphate	Mocap CE, Ethopro 10%, Mocap 15 G	I, N	Inhibits acetylcholinesterase	Ib	1, 2, 3	5.55% (1/18)
Carbofuran	Carbamate	Furadan 350, Furadan 3F, Furadam 48SC	I, N	Inhibits acetylcholinesterase	Ib	1, 3, 4	11.11% (2/18)
Methomyl	Carbamate	Lannate, Lannate 90, Lannate 21,6 SL, Lannate 40 SP, Lannate Blue	I	Inhibits acetylcholinesterase	Ib	1, 3	22.22% (4/18)
Chlorpyrifos ethyl	Organophosphate	Foley 50 CE MAX, Foley MAX 1.5%	I	Inhibits acetylcholinesterase	II	1, 3	27.77% (5/18)

^1^ Type: I = insecticide; N = nematicide. ^2^ Toxicological category (TC): Ib = very hazardous; II = moderately hazardous; III = slightly hazardous; IV = unlikely to present an acute hazard. ^3^ Criteria for inclusion in the list of highly hazardous pesticides (HHP): 1 = High; 2 = chronic effects on human health; 3 = environmental toxicity; 4 = restricted or banned by environmental conventions [7,8].

**Table 3:** Micronuclei (MN) evaluated in farmers according to the sociodemographic characteristics of the locality of San Diego Chalma in the municipality of Tehuacán, Puebla, Mexico.

Variables	Category	N	%	F			NF			*p* Value
				*n*	MN	%	*n*	MN	%	
**Total MN**	MN	**35**	**100**	**18**	**34,545**	**89.2**	**17**	**4189**	**10.8**	**0.001** *
Age	0–29 years	11	31.4	5	9718	28.1	6	223	5.3	0.001 *
	30–59 years	24	68.6	13	24,827	71.9	11	3966	94.7	
Sex	Women	9	25.7	4	7095	20.5	5	383	9.1	0.001 *
	Men	26	74.3	14	27,450	79.5	12	3806	90.9	
Birthplace	Tehuacán	19	54.3	7	13,491	39.1	12	3732	89.1	0.001 *
	San Diego Chalma	16	45.7	11	21,054	60.9	5	457	10.9	
Time of residence in the town	0–5 years	5	14.3	2	2266	6.6	3	992	23.7	0.001 *
	6–10 years	2	5.7	1	1747	5.1	1	6	0.1	
	10–20 years	7	20.0	4	5891	17.1	3	443	10.6	
	21–30 years	6	17.1	1	2956	8.4	5	846	20.2	
	>30 years	15	42.9	10	21,685	62.8	5	1902	45.4	
Education level	Elementary education	19	54.2	13	25,762	74.6	6	1403	33.5	0.001 *
	Secondary education	5	14.3	2	2976	8.6	3	1403	33.5	
	Higher Secondary education	5	14.3	2	4372	12.7	3	206	4.9	
	Technical education	3	8.6	0	0	0	3	992	23.7	
	No education	3	8.6	1	1435	4.1	2	185	4.4	
Occupation	Farmer	18	51.4	18	34,545	100	0	0	0	0.001 *
	Businessman	10	28.6	0	0	0	10	2049	48.9	
	Housewife	2	5.7	0	0	0	2	1377	32.9	
	Student	3	8.6	0	0	0	3	727	17.4	
	Minors	2	5.7	0	0	0	2	36	0.8	
Smoker	1–5 cigarettes/week	9	25.7	7	14,694	42.5	2	793	18.9	0.001 *
	No smoking	26	74.3	11	19,851	57.5	15	3396	81.1	
Alcohol consumer	2 to 3 times a week	2	5.7	1	2702	7.9	1	625	14.9	0.001 *
	Once a week	16	45.7	11	22,195	64.2	5	1080	25.8	
	No alcohol	17	48.6	6	9648	27.9	11	2484	59.3	
Diseases Infections	Once a year	9	25.7	5	5824	16.9	4	734	17.5	0.280
	None	26	74.3	13	28,721	83.1	13	3455	82.5	

F: People of farmers; NF: People of non-farmer; N = total population of farmer; *n* = population per group of study; % = percentage; MN = number of micronuclei. * Significant statistical differences (*p* ≤ 0.05).

**Table 4:** Comparison of BMCA results between exposed (F) and control (NF) groups.

Variables	Median		IQR	Mann–Whitney U Test	*Z*	1–*β*	*d*	Significance *p* ≤ 0.05
MNi	(F)=	714	164	2,600,576	−102.24	0.89	6.17	0.001
	(NF)=	36	23					
CC	(F)=	502	245	0	−106.07	0.68	4.27	0.001
	(NF)=	0	29					
KR	(F)=	206	270	8,692,340	−93.37	0.96	1.76	0.001
	(NF)=	0	34					
BN	(F)=	223	165	2,405,619	−102.53	0.91	2.66	0.001
	(NF)=	2	56					
KL	(F)=	2096	1351	0	−106.07	0.94	2.88	0.001
	(NF)=	0	99					
PY	(F)=	1129	766	1,834,096	−103.38	0.83	2.65	0.001
	(NF)=	0	78					
MN	(F)=	2581	671	0	−106.05	0.68	3.39	0.001
	(NF)=	499	955					
BC	(F)=	2792	1904	757,872	−104.94	0.99	13.86	0.001
	(NF)=	9501	339					

F: People of farmers; NF: People of non-farmer; BMCA = buccal micronucleus cytome assay. Note: Total number of micronuclei (MNi), condensed chromatin (CC), karyolysis (KL), binucleate cells (BN), micronuclei (MN), karyorrhexis (KR), pyknosis (PY), basal cells (BC). IQR = interquartile range; 1–*β* = Power (probabilistic error) of a study; *d* = Cohen effect size. Bilateral significance according to Mann-Whtiney U test (*p* ≤ 0.05).

**Table 5:** Socio-demographic characteristics in the development of Micronuclei (MN) evaluated in no farmers of the locality of San Diego Chalma in the municipality of Tehuacán, Puebla, Mexico.

Predictor Variable	^1^ B	^2^ Odds Ratio [Exp B]	95% CI		*p* Interact
			Lower	Lower	
Groups					
NF	−5.570				0.001
F	0	1			
Age					0.001
30–59 years	2.801	16.464	14.315	18.935	0.001
0–29 years	0	1			
Birth place					0.001
Tehuacán	−1.476	0.229	0.217	0.240	0.001
San Diego Chalma	0	1			
Sex					0.001
Men	0.870	2.386	2.123	2.681	0.001
Women	0	1			

F: People of farmers; NF: People of non-farmer; −2 Log-likelihood = 1122.528. Hosmer and Lomeshow googness-of-fit test (*p* = 0.085). ^1^ B: coefficient of predictor variable; ^2^ Odds Ratio [Exp B]: multivariate risk coefficient.

**Table 6:** Probability forecasting in the development of Micronuclei (MN) cells with respect to sociodemographic characteristics.

Variables				N (TMN = 38,734)	PFMN	
Birthplace	Age	Sex	People		*n*	%
Tehuacán	0–29 years	Men	NF	96	217.4	3.3
			F	6475	6353.6	96.7
		Women	NF	91	1.3	1.4
			F	0	89.7	98.6
	30–59 years	Men	NF	3253	3183.4	36.0
			F	5581	5650.6	64.0
		Women	NF	292	329.8	19.1
			F	1435	1397.1	80.9
San Diego Chalma	0–29 years	Men	NF	36	3.2	0.2
			F	1747	1779.8	99.8
		Women	NF	0	1.1	0.1
			F	1496	1494.9	99.9
	30–59 years	Men	NF	421	402.1	2.9
			F	13,647	13,666.1	97.1
		Women	NF	0	50.7	1.2
			F	4164	4113.3	98.8

N = Micronuclei (MN) cells per predictor variable. TMN = Total Micronuclei (MN) cells. PFMN: Micronuclei (MN) cells forecast probability.

## Data Availability

The data presented in this study are available on request from the corresponding author.
